# Commencement American Veterinary College

**Published:** 1886-04

**Authors:** 


### Proceedings Veterinary Medical Societies.

### COMMENCEMENT AMERICAN VETERINARY COLLEGE.

The eleventh annual commencement of the American Veterinary College was held at Chickering Hall, New York City, on Monday evening, March 1. Twenty-eight young men formed the graduating class, and had the degree of Doctor of Veterinary Surgery conferred upon them by the President, Samuel Marsh. The valedictory was delivered by Robert Corwin Jones. Addresses were made by several of the trustees. The following is a list of the graduates: Messrs. D. C. Ashley, of Massachusetts, E. B. Buckley, of Connecticut, M. L. Bignell, of Pennsylvania, S. Bradley, of New York, C. E. Bridge, of Pennsylvania, J. S. Candee, of New York, J. J. Cattanach, of New York,

E. A. Child, of Florida, A. J. Dodin, of New York, Th. H. Doyle, of New York, F. H. Flagge, of New York, F. W. Hopkins, of Ireland, R. C. Jones, of New York. C. Kuenhe, Ph. G., of New Jersey, W. J. Magee, of New York, B. F. Munich, of Pennsylvania, F. W. Meyer, of Pennsylvania, Maurice O'Connell, of Massachusetts, A. W. Radley, of Pennsylvania, A. K. Robertson, of Iowa, H. Schmidt, Jr., of Ohio, A. Strang, of New Yok, M. R. Trumbower, of Illinois, G. G. Vanwater, of New York, G. L. Warner, of New York, J. A. Wolwrath, of New York, R. Weir, of Mass, A. C. Young, of Utah. The prizes were carried off by Dr. G. L. Warner the gold medal of the Board of Trustees; Dr. A. Strange, that of the N. Y. State Veterinary Society; Dr. A. C. Young, the prize of the Alumni Association; Dr. F. W. Hopkins, and Mr. J. D. Fair, the anatomical prizes.

